# Macrophage, the potential key mediator in CAR-T related CRS

**DOI:** 10.1186/s40164-020-00171-5

**Published:** 2020-07-10

**Authors:** Zhaonian Hao, Ruyuan Li, Li Meng, Zhiqiang Han, Zhenya Hong

**Affiliations:** 1 The Second Clinical School Affiliated Tongji Hospital, Tongji Medical College, Huazhong University of Science and Technology, Wuhan, 430030 Hubei China; 2 Department of Hematology, Tongji Hospital, Tongji Medical College, Huazhong University of Science and Technology, No.1095 Jie Fang Avenue, Hankou, Wuhan, 430030 Hubei China; 3 Cancer Biology Research Center (Key Laboratory of the Ministry of Education), Tongji Hospital, Tongji Medical College, Huazhong University of Science and Technology, No.1095 Jie Fang Avenue, Hankou, Wuhan, 430030 Hubei China

**Keywords:** Chimeric antigen receptor T cell therapy, Cytokine release syndrome, Macrophage

## Abstract

Chimeric antigen receptor (CAR) T cell therapy is a new frontier in cancer therapy. The toxicity of cytokine release syndrome (CRS) has become one of the major challenges that limits the wider use of CAR T cells to fight cancer. Exploration of CRS pathogenesis and treatment is becoming the main focus of ongoing studies. Myeloid-derived macrophages were found to play a critical role in CRS pathogenesis, and these cells mediate the major production of core cytokines, including IL-6, IL-1 and interferon (IFN)-γ. Colocalization of macrophages and CAR T cells was also identified as necessary for inducing CRS, and CD40L-CD40 signaling might be the key cell–cell interaction in the tumor microenvironment. Macrophages might also take part in endocrine and self-amplified catecholamine loops that can directly activate cytokine production and release by macrophages during CRS. In addition to tocilizumab and corticosteroids, several novel CRS therapies targeting macrophage-centered pathways have shown much potential, including GM-CSF blockade and administration of atrial natriuretic peptide (ANP) and α-methyltyrosine (metyrosine, MTR). In the present review, we summarized the role of macrophages in CRS and new developments in therapeutic strategies for CRS-associated toxicities.

## Background

Chimeric antigen receptor (CAR) T cell therapy is one of the greatest innovations in fighting tumors. The remarkable success of CD19-targeting CAR-mediated treatment of refractory B cell malignancies is a key first step in overcoming cancers. However, side reactions have become one of the major barriers to applying CAR therapy for tumor treatment, and cytokine release syndrome (CRS) is common and even life-threatening. CRS was previously described as a clinical syndrome featuring overactivation of the immune system associated with CAR T cell expansion and an increase in serum cytokines and proinflammatory substances [1, 2]. The severity of CRS can be classified from grade 1 to 4, with manifestations from fever and hypotension to hypoxia, shock and organ toxicities [3]. Currently, the pathophysiology of CRS is not completely understood, but it is commonly considered to occur due to the on-target effects of CAR T cells and somehow triggers activation of bystander cells (both immune cells and nonimmune cells) in the tumor environment [4]. Activation of these bystander cells initiates the proinflammatory process and the massive production of cytokines, which eventually causes CRS. Cytokines, including IL-6, IL-10 and interferon (IFN)-γ, have been confirmed to be the core cytokines and are consistently found to be elevated in the serum of patients with CRS [5–8]. Despite our limited understanding of the mechanism by which the cascade of the immune response is initiated and amplified, ultimately leading to cytokine storms, identifying these core cytokines can provide promising clues that may ultimately determine the contributors to CRS pathogenesis. The complex composition of the CAR T cell-associated tumor microenvironment makes the tracing difficult. To date, studies on the mechanisms of CRS have suggested that myeloid-derived macrophages are potential key regulators of the pathogenesis of CRS.

In this review, we summarize the current progress in CRS mechanism research and propose that myeloid-derived macrophages might be the major source of the core cytokine IL-6 and the key mediator of the initiation and exacerbation of immune overresponse that disrupts counterregulatory homeostatic mechanisms and leads to cytokine storms.

### Macrophages might be the major source of the core cytokine in CRS

**Fig.1:**
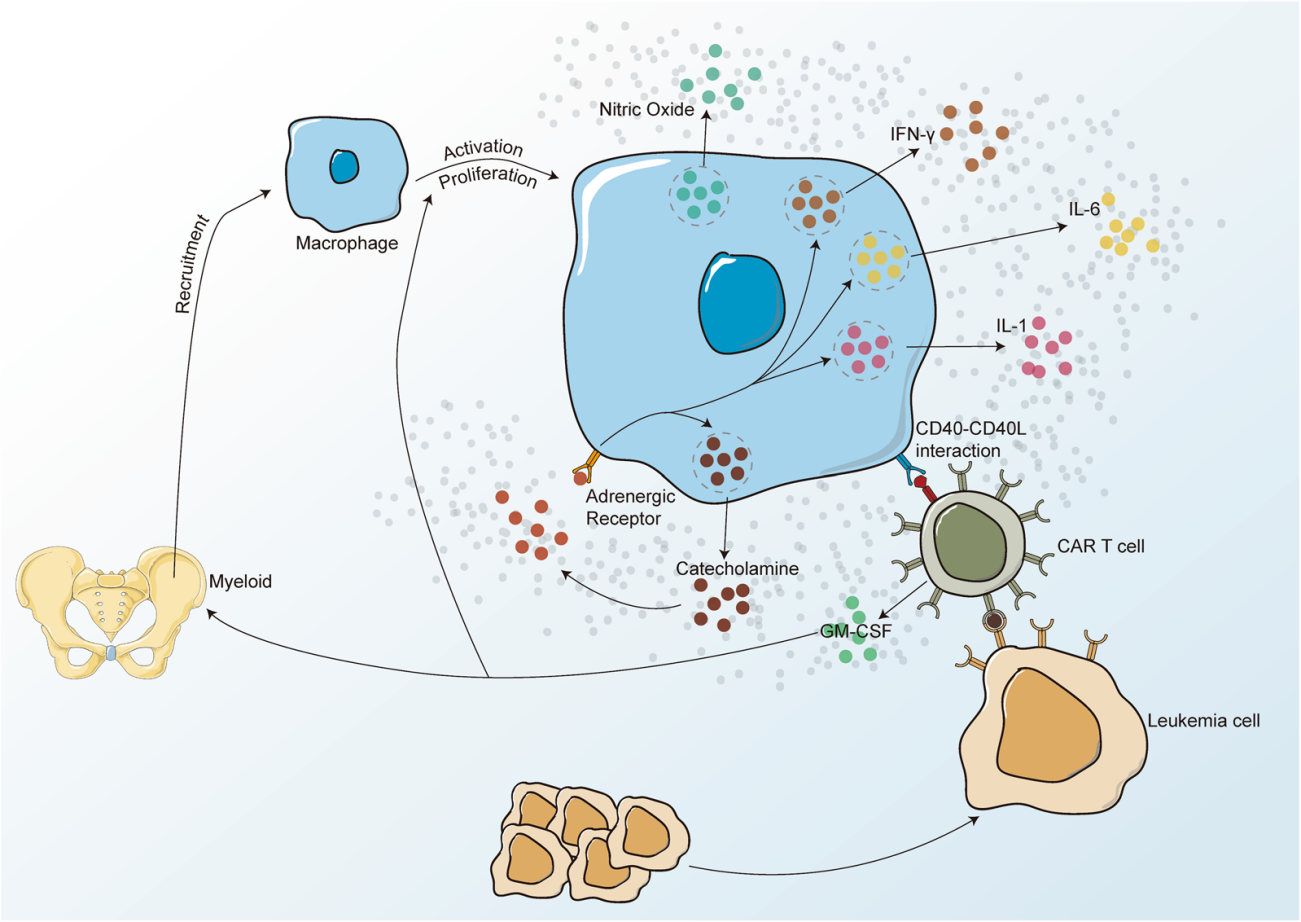
Illustration of the role of macrophage in CRS**.** The CRS-related macrophage is mainly derived from myeloid. In CAR-T scenario, the infusion of CAR T cells initiated the mobilization of monocyte-macrophage lineage in myeloid. Once recruited to the colocalization of CAR T cells and tumor cells, the macrophages turned to activation and proliferation. The core cytokines in CRS were then secreted from the macrophage to the microenvironment, which was conducted by multiple mechanisms including the CD40-CD40L interaction with CAR T cells and a self-enhanced loop of catecholamine. And all these cytokines were then participating in the genesis and upsurge of CRS. Also, the underlying production of GM-CSF from CAR T cells was proved able to stimulate and enhance the mobilization and proliferation of monocyte-macrophage lineage, which unsurprisingly led to a self-amplified loop of the whole process of CRS. *IFN* interferon, *GM-CSF* Granulocyte-macrophage Colony Stimulating Factor, *CAR* chimeric antigen receptor

The pathophysiology of CRS is not completely understood. CRS can be triggered by on-target effects induced by the binding of an engineered T cell to its target and subsequent recruitment and activation of bystander immune or nonimmune cells. An increasing number of reports have revealed that myeloid-derived macrophages play a critical role in CRS pathogenesis. Figure 1 summarizes the current understanding of the role of macrophages in CRS.

IL-6, IL-10, and IFN-γ are presently regarded as the core cytokines involved in CAR T cell-related CRS [4, 9]. Elevated levels of these core cytokines have been confirmed in patients with CRS and in murine models. Inhibitors such as tocilizumab that block the core cytokines have also been approved as therapeutic strategies to manage CRS in the clinic [8, 10, 11]. Tracking down the origin of these core cytokines would identify the onset of CRS.

At present, focusing on the production of IL-6 is a logical choice for researchers to uncover the CRS mechanism. IL-6 has been confirmed as a cytokine with pleiotropic utility [12, 13] that is secreted under different conditions, such as stress, infection, and tissue injuries. IL-6 is released from not only immune cells but also vascular endothelial cells, mesenchymal cells, fibroblasts and others through stimulation of Toll-like receptors (TLRs) or through IL-1 or tumor necrosis factor (TNF)-α [14]. During CRS, both IL-6 and its downstream effectors play important roles in the development of clinical symptoms. High levels of IL-6 could lead to vascular leakage and activation of the complement, coagulation cascade, which induces disseminated intravascular coagulation and myocardial dysfunction [8]. Previous studies on CRS reported that IL-6 is mainly produced by activated T cells [15, 16]. Other researchers have revealed that endothelial cells in vessels and the monocyte-macrophage lineage also participate in IL-6 production in CRS [17, 18]. Understanding the source, signaling and cellular targets of IL-6 is paramount to informing the design of clinical studies.

Within the huge advantages it had in the novel xenogeneic model of CRS, it is easier to identify the origin of the population in the tumor environment the CRS. Several studies mentioned noteworthy tumor-infiltrating myeloid cells, including neutrophils, eosinophils, dendritic cells and macrophages, in the tumor site. IL-6 expression was suggested to mainly be the result of CRS-associated macrophages in a severe combined immunodeficiency (SCID)-beige mouse model. Not surprisingly, analysis of cell surface markers on these CRS-associated macrophages showed a high level of Ly6C, indicating a more proinflammatory lineage of monocyte-macrophage. Moreover, blocking IL-6 with its receptor antagonist tocilizumab could abate CRS by several mechanisms [10, 19, 20]. Myeloid-derived macrophages seemed to have more important functions in CRS than we previously expected, based on accumulating evidence [21–23]. Bondanza et al. used another NOD/SCID/IL2ry^null^ (NSG) mouse model and found that monocytes were the major source of IL-6 in CRS [11], and depletion of monocytes prevented mice from CRS-associated features. The number of monocytes was also negatively correlated with survival. However, they found that monocyte suppression had a negative impact on CAR T cell expansion and leukemic clearance, which is paradoxical with the findings of other studies.

Interleukin‑1, a major mediator of local and systemic inflammation, is also known as a core cytokine in the process of CRS [4, 24]. In the same SCID-beige model mentioned above, Sadelain et al. suggested that the type 1 IL-1 receptor (IL1R1) was upregulated in tumor-associated myeloid cells, while the type 2 IL-1 receptor (IL1R2) was increased in splenic myeloid cells outside the tumor bed. Given that ILR2 serves as a decoy receptor to weaken the effect of ILR1 in inflammation, the team hypothesized that endogenous inhibition of IL-1 is not sufficient to restrain the proinflammatory mediation of IL-1. As expected, the use of anakinra, an IL-1 receptor antagonist, abrogated CRS-related mortality [24].

Canonically activated macrophages, also known as M1 macrophages, act as proinflammatory mediators. Inducible nitric oxide synthase (iNOS) is one of the proinflammatory cytokines expressed by M1 macrophages. The participation of iNOS in CRS has been confirmed in recent studies. Sadelain et al. confirmed that macrophages were the major source of iNOS after administration of CAR T cells in vivo [10]. Aberrant NO production leads to vasodilation and hypotension [25], which is the main life-threatening clinical manifestation during CRS that is caused by CAR T administration. Moreover, treatment with the iNOS inhibitor L-NIL or 1400 W could improve survival and alleviate toxicity under conditions of severe CRS in the study by Sadelain. On the other hand, there is also evidence suggesting other mechanisms by which iNOS participates in CRS. George et al. found that IL-6 could induce Nos2 transcription in plasma cells [26]. IL-1β could also induce gene expression and synthesis of iNOS [27]. As IL-6 and IL-1 significantly contribute to CRS, it is believed that iNOS has a role in CRS. Blocking both IL-1 and IL-6 alleviates severe CRS by reducing the iNOS + macrophage fraction equivalently, suggesting that NO is one unifying downstream effector of IL-1 and IL-6.

GM-CSF is produced by activated hematopoietic and nonhematopoietic cells, acts as an important mediator during inflammatory responses and interacts with myeloid populations, including monocytes and macrophages [28, 29]. It is well known that GM-CSF can activate macrophages by enhancing their responsiveness to CSF-1 (macrophage CSF) [29]. In addition, the GM-CSF receptor has been shown to be highly expressed in microglia, brain macrophages, and astrocytes. Thus, these neuro cells can also respond to inflammation, which may explain the neuro-associated symptoms after CAR T cell therapy [30]. In addition, GM-CSF is involved in a sophisticated network of inflammatory mediation. Cytokines such as IL-1β and tumor necrosis factor can act as both products of GM-CSF and mediators of its downstream effectors [28]. Given that monocytes and cytokines play a key role in CRS, blocking GM-CSF may be a potential strategy in the clinical management of CRS. Recent studies have confirmed the considerable participation of GM-CSF in CRS. A high expression level of GM-CSF was observed in a patient-derived xenograft model and preclinical models during the CRS stage [10, 22]. It is believed that CART T cells are the major source of GM-CSF [21, 22], which was further verified through CRISPR knockout technology in a study by Sterner and colleagues. The group found that knocking out GM-CSF during CAR T cell manufacture could reduce the level of GM-CSF in a xenograft model [22]. To investigate the effect of blocking GM-CSF, they tested lenzilumab, a monoclonal antibody that neutralizes human GM-CSF, in a patient-derived xenograft model. A significant reduction in CRS-induced weight loss, as well as cytokines such as IL-2 and IL-1Ra, was observed. Moreover, brain MRIs also showed decreased brain inflammation. Another team used a neutralization antibody (Ab) against GM-CSF and found similar results [21]. In a study by Sachdeva, proinflammatory cytokines, such as IL-6, MCP-1, and IL-8a, were significantly reduced by the Ab in a dose-dependent manner. At the same time, the Ab has no effect on the expression levels of key T cell cytokines, such as IFN-γ, TNF-α and IL-10, even at higher doses. This result suggests that neutralizing GM-CSF does not affect the effect of CAR T cells. Given that early prophylactic tocilizumab treatment could increase the overall rates of neurotoxicity in clinical trials [19, 31], GM-CSF may be the next potential target and prospect in CRS treatment. All-in-one CAR T cells, which knock out GM-CSF, could be an alternative CAR T cell design.

Ferritin, a universal intracellular protein that stores iron and releases it in a controlled fashion, was confirmed at an elevated level in the serum of patients with severe CRS patients [7, 9, 32, 33]. It is well known that serum ferritin is an acute-phase reactant that indicates the degree of inflammation in infection, rheumatosis, hematonosis and malignancy. Experiments have revealed that high levels of ferritin are released in response to macrophage activation resulting from IFN stimulation or coculture with pathogens. Clinical observations of hemophagocytic lymphohistiocytosis (HLH) and macrophage activation syndrome (MAS) in children reported that ferritin was also elevated in MAS/HLH, which resembled the increase in ferritin in CRS [34, 35]. The similar pattern of macrophage activation, including elevated ferritin, low fibrinogen and the cytokine profile, indicate a possible parallel pathophysiology of macrophage involvement [32].

### Macrophages play a key role in the CAR T cell-associated tumor microenvironment during CRS pathogenesis

Evidence has shown that the IL-6 increase during CRS was specifically identified in sites at which CAR T cells colocalized with tumor tissue. Giavridis and colleagues infused mCD40L-expressing CAR T cells in the NSG model, and a CD40L-CD40 interaction between CAR T cells and monocyte-macrophage lineage was identified during CRS, which also led to more severe CRS [10]. Given that IL-6 production can be directly induced by CD40L signaling [36], there might be a CAR T cell-macrophage regulatory axis through CD40L-CD40 signaling associated with cytokine production. However, since hCD40L cannot directly interact with mCD40 [37], the occurrence of CRS does not seem to be affected by the absence of the CD40L-CD40 axis in the mouse model that is commonly used in studies. It is not determined whether the direct interaction of CAR T cells and macrophages is required for CRS. However, this direct interaction pattern is one of the important regulatory mechanisms of cytokine production in CRS.

Previous studies have reported that macrophages, which are the major sources of inflammatory cytokines, can respond to and secrete catecholamines by activating adrenergic receptors when exposed to inflammatory stimuli [38, 39]. This in turn causes massive cytokine production in macrophages, as revealed in lung injury and autoimmune encephalomyelitis models [39, 40]. Strikingly, the catecholamine surge was discovered during CRS, and the hCART19-Raji cell interaction was confirmed to lead to the release of catecholamines and cytokines (IL-2, TNF, IFN-γ, and MIP-1α). Experiments have confirmed that α1-adrenergic receptor-dependent signaling is responsible for activation of the downstream production of many cytokines, including IL-6, TNF, and MIP-2, and catecholamines in macrophages in a study by Staedtke. The production of catecholamine was identified as a tyrosine hydroxylase (TH) dependent pattern that could be diminished by α-methyltyrosine (metyrosine, MTR), then the massively secreted catecholamines in turn interact with the macrophages to form a self-amplification loop [23]. These novel findings by Staedtke suggest that catecholamines can enhance inflammatory injury during CRS through a self-amplifying feed forward loop in macrophages.

### Diagnosis, grading, and current treatment of CRS

Since CRS is thought to represent an inflammatory cytokine surge, identifying inflammatory factors and circulating cytokines can be helpful and serve as biomarkers in CRS diagnosis and classification of severity. CRP is generally accepted as a canonical biomarker of CRS due to its correlation with CRS, and measurement of CRP is rapid and easy [16]. A subsequent study confirmed a higher level of CRP in individuals with CRS grade ≥ 4 than in those with grade 0–3 [2]. Similar to CRP, baseline ferritin was observed to be elevated in CRS, and the peak level of ferritin was also increased in grade ≥ 4 CRS [3, 32]. Measurements of peak levels of cytokine profiles have identified several cytokines that correlate with CRS severity, including IFN-γ, IL-6, IL-8, IL-10, IL-15, sIL2Rα, sgp130, sIL6R, MCP-1, MIP-1α, MIP-1β, TNFRp55 and GM-CSF. Additionally, some of these cytokines were increased in the first 36 h after infusion of CAR T cells, including IFN-γ, IL-6, IL-8, IL-10, IL-15, MCP-1, TNFRp55, and MIP-1β, and demonstrated similar kinetic patterns in B-ALL, CLL, and NHL patients, suggesting that these cytokines may be used to predict severe CRS (grade ≥ 4) [2, 32]. Other clinical indices, such as ALT, AST, BUN, LDH and Cr, showed a significant increase in grade ≥ 4 CRS versus grade 0–3 CRS, but unfortunately, none of these factors were useful for predictions in the early period of CRS onset. Additionally, low fibrinogen and prolonged coagulation were significantly associated with grade ≥ 4 CRS in the pediatric cohort, while hypofibrinogenemia and mild coagulopathy were detected in the adult cohort [32]. The current biomarkers of CRS are summarized in Table 1.

**Table 1:** Biomarkers for CRS predication

Biomarkers	Grade 0–3	Grade ≥ 4
Inflammatory factor		
CRP	↑	↑↑
Ferritin	↑	↑↑
Cytokine profile		
IFN-γ^a^^,^^b^	↑	↑↑
IL-6^a^^,^^b^	↑	↑↑
IL-8^a^^,^^b^	↑	↑↑
IL-10^a^	↑	↑↑
IL-15^a^	↑	↑↑
sIL2Rα^b^	↑	↑↑
sIL6R^b^	↑	↑↑
MCP-1^a^^,^^b^	↑	↑↑
MIP-1α^b^	↑	↑↑
MIP-1β^a^^,^^b^	↑	↑↑
GM-CSF^b^	↑	↑↑
TNFRp55^a^	↑	↑↑
sgp130^b^	↑	↑↑
Blood biochemistry		
ALT	↑	↑↑
AST	↑	↑↑
BUN	↑	↑↑
LDH	↑	↑↑
Cr	↑	↑↑
Coagulation		
Fibrinogen (Pediatric)	–	↓
Fibrinogen (Adult)	↓	↓
PT (Pediatric)	–	↑
PT (Adult)	↑	↑
APTT (Pediatric)	–	↑
APTT (Adult)	–	–

^a^Cytokines that helpful in CRS prediction can be assessed within 36 h post CAR T cells infusion

^b^Peak level of cytokines in the first month after CAR T cells infusion that are associated with more severe CRS

Fever or elevations in the aforementioned biomarkers may indicate impending CRS in patients receiving CAR T cell therapy. These patients should be closely monitored and assessed for CRS severity. The most widely used grading assessment was developed by the National Cancer Institute Common Terminology Criteria for Adverse Events (CTCAE) (Table 2). Given that immunosuppression therapies are heavily associated with the treatment algorithm of CRS, additional diagnoses should be performed to rule out other causes of systemic inflammatory response, including infection and malignancy progression.

**Table 2:** Grading and management of CRS

Grade	Grading assessment		Treatment^a^		
	Modified CTCAE 4.0^b^	CTCAE 5.0 [41]^,c^	Anti-IL-6 Therapy	Corticosteroids	Additional supportive care
Grade 1	Symptoms are not life threatening and require symptomatic treatment only Fever, nausea, fatigue, headache, myalgias, malaise	Fever (≥ 38 °C)	For prolonged CRS (> 3 days) in patients with significant symptoms and/or comorbidities, consider tocilizumab as per Grade 2	N/A	Empiric broad-spectrum antibiotics, consider granulocyte colony-stimulating factor (G-CSF) if neutropenic Maintenance IV fluids for hydration Symptomatic management of organ toxicities
Grade 2	Symptoms require and respond to moderate intervention Oxygen requirement < 40% or Hypotension responsive to fluids or low dose of one vasopressor or Grade 2 organ toxicity	Fever with hypotension not requiring vasopressors and/or Hypoxia requiring low-flow nasal cannula or blow-by	Tocilizumab 8 mg/kg IV over 1 h (not to exceed 800 mg/dose) Repeat in 8 h if no improvement; no more than 3 doses in 24 h, with a maximum of 4 doses total	For persistent refractory hypotension after 1–2 doses of anti-IL-6 therapy: Dexamethasone 10 mg IV every 6 h (or equivalent)	IV fluid bolus as needed For persistent refractory hypotension after two fluid boluses and anti-IL-6 therapy: Start vasopressors, consider transfer to intensive care unit (ICU), consider echocardiogram, and initiate other methods of hemodynamic monitoring Manage per Grade 3 if no improvement within 24 h after starting anti-IL-6 therapy Symptomatic management of organ toxicities
Grade 3	Symptoms require and respond to aggressive intervention Oxygen requirement ≥ 40% or Hypotension requiring high dose^d^ or multiple vasopressors or Grade 3 organ toxicity or grade 4 transaminitis	Fever with hypotension requiring a vasopressor with or without vasopressin and/or Hypoxia requiring high-flow cannula, face mask, nonrebreather mask, or Venturi mask	Anti-IL-6 therapy as per Grade 2 if maximum dose not reached within 24 h period	Dexamethasone 10 mg IV every 6 h (or equivalent). If refractory, manage as grade 4	Transfer to ICU, obtain echocardiogram, and perform hemodynamic monitoring Supplemental oxygen. IV fluid bolus and vasopressors as needed Symptomatic management of organ toxicities
Grade 4	Life-threatening symptoms Requirement for ventilator support or Grade 4 organ toxicity (excluding transaminitis)	Fever with hypotension requiring multiple vasopressors (excluding vasopressin) and/or Hypoxia requiring positive pressure (eg, CPAP, BiPAP, intubation and mechanical ventilation)	Anti-IL-6 therapy as per Grade 2 if maximum dose not reached within 24 h period	Dexamethasone 10 mg IV every 6 h (or equivalent). If refractory, consider methylprednisolone 1000 mg/day IV	ICU care and hemodynamic monitoring Mechanical ventilation as needed IV fluid bolus and vasopressors as needed Symptomatic management of organ toxicities

*CTCAE* Common Terminology Criteria for Adverse Events, *BiPAP* bilevel positive airway pressure, *CPAP* continuous positive airway pressure therapy, *IV* intravenous

^a^CCN Clinical Practice Guidelines in Oncology, Management of Immunotherapy‑Related Toxicities (Version 1.2020)

^b^Revised CRS grading system based on CTCAE v4.0 by Lee et al. [3]

^c^Fever is defined as temperature > 38 °C not attributable to any other cause. In patients who have CRS then receive antipyretics or anti-cytokine therapy such as tocilizumab or steroids, fever is no longer required to grade subsequent CRS severity. In this case, CRS grading is driven by hypotension and/or hypoxia

^d^see specific definition of high-dose vasopressors [42]

In addition to the early recognition and grading of CRS, treatment of the associated toxicities is a crucial challenge in CAR administration. The current strategy for treating CRS is based on the experience of clinical physicians and expert opinions [3, 31]. The approach for a specific patient depends on the severity of CRS according to the grading scheme and revised version developed by the National Cancer Institute (NCI) [3, 41]. Detailed treatments according to the National Comprehensive Cancer Network (NCCN, Version 1 2020) is shown in Table 2. Tocilizumab and corticosteroids are routinely recommended for patients with grade 2–4 CRS.The increase in serum IL-6 has been observed in CRS patients following CAR T cell therapy. Tocilizumab was considered an appropriate treatment for severe CRS, which was confirmed in studies showing that tocilizumab was able to rapidly reverse the symptoms of CRS [3, 43, 44]. In August 2017, the FDA approved tocilizumab for the treatment of CRS in patients 2 years of age or older.

Clinical evidence demonstrates that corticosteroids can effectively abate CRS, and corticosteroids are a preferred choice for controlling CRS toxicities due to the efficacy of immunosuppression. Corticosteroids were previously avoided as a first-line treatment for CRS owing to the known effects of tocilizumab, which rapidly alleviates symptoms, and the possible adverse effects of corticosteroids on the immune system [16]. Because of their improved penetration of the blood brain barrier, steroids are therefore recommended for controlling neurotoxicity [45]. In addition, tocilizumab is a monoclonal antibody drug that can specifically eliminate IL-6 in the circulation without any adverse effects on central nervous system (CNS) IL-6 signaling because it cannot cross the blood brain barrier. According to the observations from the first clinical trials of blinatumomab, a prophylactic regimen in which corticosteroids are administered before cytoreduction, showed a prominent effect of corticosteroids on CRS prevention [46, 47]. Two recent clinical trials revealed that the application of corticosteroids did not affect the proliferation, duration or the antitumor effects of CAR T cells [48, 49]. One possible explanation was that a high dose of steroids for a shorter term (average 4 days; 91.3% ≤ 7 days) was used in a study by Liu [48]. To date, the National Comprehensive Cancer Network (NCCN) guidelines (version 1 in 2020) suggest that corticosteroids are preferred for cases with persistent refractory hypotension after IL-6 blockade and in the presence of severe CRS.

### Macrophage-centered outlooks on CRS treatment

Previous studies have identified IL-1 as one of the core cytokines associated with CRS. Anakinra, an IL-1R antagonist that is able to ameliorate inflammation [24], was confirmed to be effective in CRS treatment. Giavridis and colleagues designed and constructed IL-1R antagonist-secreting CAR T cells and found significant prevention of CRS-related mortality. The monocyte-macrophage lineage is thought to produce IL-1 upon activation, and IL-6 is produced in response to IL-1 signaling. Exploration of the protective mechanisms of IL-1 blockade and IL-6 blockade has revealed that blocking both signals can similarly reduce iNOS + macrophage fractions and ameliorate CRS. However, combined blockade of IL-1 and IL-6 did not further decrease the fraction of iNOS + macrophages, which indicated a common pathway of IL-6 and IL-1 in CRS [10]. Additionally, application of the iNOS inhibitor L-NIL or 1400 W could improve survival and alleviate toxicity in severe CRS. Neutralizing GM-CSF could be an alternative strategy for managing CRS, as GM-CSF is an important mediator of monocytes in the inflammatory response. Sterner and colleagues proved the effect of lenzilumab on reducing proinflammatory cytokines, as well as neurotoxicity [22]. Neutralizing antibodies against GM-CSF also had similar effects in a dose-dependent manner [21].

To date, only tocilizumab has been approved by the FDA for CRS treatment. Anakinra, approved for use in active rheumatoid arthritis, is undergoing clinical trials for CRS treatment and verification of its efficacy is still pending (NCT04359784, NCT04148430, and NCT04150913). Other potential agents still need to be verified further in preclinical models and clinical trials. There have also been other attempts to perform clinical trials in this field (accessed June 4, 2020), as shown in Table 3.

**Table 3:** The Undergoing clinicals trails of CRS management

NCT no	Trails	Status	Conditions	Inventions	Locations	Phase
NCT04071366	A study of itacitinib for the prevention of cytokine release syndrome induced by immune effector cell therapy	Not yet recruiting	Cytokine release syndrome	Drug: Itacitinib Drug: Immune effector cell therapy	Univ. of Miami Sylvester Comprehensive Cancer Center, Miami, Florida, United States Moffitt Cancer Center, Tampa, Florida, United States Massachusetts General Hospital Boston, Massachusetts, United States (and 7 more…)	Phase 2
NCT03533101	Tocilizumab for cytokine release syndrome prophylaxis in haploidentical transplantation	Unknown	Cytokine release syndrome stem cell transplant complications	Drug: Tocilizumab	Hospital Universitario Dr. Jose E Gonzalez UANL, Monterrey, Nuevo Leon, Mexico	Phase 1 Phase 2
NCT04048434	Extracorporeal cytokine adsorption as additive treatment of CAR-T associated Cytokine Release Syndrome (CRS)	Not yet recruiting	Cytokine Release Syndrome CAR-T	Device: Cytosorb		Not applicable
NCT04082910	Metoprolol for the treatment of cytokine release syndrome in patients treated with chimeric antigen receptor T cells	Recruiting	Solid tumor Hematologial malignancy	Drug: Metoprolol Drug: anti-TNFα antibody	Biotherapeutic Department of Chinese PLA General Hospital Beijing, Beijing, China	Phase 1 Phase 2
NCT04359784	Anakinra for the prevention of cytokine release syndrome and neurotoxicity in patients with B-cell lymphoma receiving CD19-targeted CAR-T cell therapy	Not yet recruiting	B-cell non-hodgkin lymphoma	Biological: Anakinra Biological: Axicabtagene Ciloleucel	Fred Hutch/University of Washington Cancer Consortium, Seattle, Washington, United States	Phase 2
NCT03755414	Study of itacitinib for the prophylaxis of graft-versus-host disease and cytokine release syndrome after T-cell replete haploidentical peripheral blood hematopoietic cell transplantation	Recruiting	Acute myelogenous leukemia Acute lymphocytic leukemia Myelodysplastic syndromes Non-hodgkin lymphoma Hodgkin disease	Procedure: Stem cell transplantation Drug: Itacitinib Other: Functional Assessment of Cancer Therapy-Bone Marrow Transplant (FACT-BMT) Other: Human Activity Profile	Washington University School of Medicine, Saint Louis, Missouri, United States	Phase 1
NCT02906371	Study of the tocilizumab optimization timing for CART19 associated cytokine release syndrome	Active, not recruiting	Lymphoblastic leukemia, acute, childhood	Drug: Tocilizumab Biological: CART 19	Children's Hospital of Philadelphia, Philadelphia, Pennsylvania, United States	Not Applicable
NCT04048525	Cytokine removal with CVVHD compared to CVVH	Completed	Cytokine release syndrome Sepsis AKI	Procedure: CVVHD Procedure: CVVH	Hospital Universitari de Bellvitge, L'Hospitalet de Llobregat, Barcelona, Spain Hospital de la Santa Creu i Sant Pau, Barcelona, Spain	Not Applicable
NCT02007239	Tocilizumab and Hemophagocytic Lymphohistiocytosis (HLH)	Active, not recruiting	Hemophagocytic lymphohistiocytosis	Drug: tocilizumab	Children's Hospital of Philadelphia, Philadelphia, Pennsylvania, United States	Phase 2
NCT04148430	A study of anakinra to prevent or treat severe side effects for patients receiving CAR-T cell therapy	Recruiting	B cell ALL B-cell lymphoma B-cell non hodgkin lymphoma	Drug: Anakinra	Memorial Sloan Kettering Cancer Center New York, New York, United States	Phase 2
NCT03275493	Humanized CD19 CAR-T cells with CRS suppression technology for r/r CD19 + acute lymphoblastic leukemia	Recruiting	Acute lymphoblastic leukemia CD19 positive relapse refractory	Biological: Humanized CD19 CAR-T cells Biological: Humanized CD19 CAR-T cells with CRS suppression technology	The first affiliated hospital of soochow university Suzhou, Jiangsu, China	Phase 1 Phase 2
NCT04150913	Anakinra in CAR-T cell mediated neurotoxicity	Not yet recruiting	Non hodgkin lymphoma Refractory non-hodgkin lymphoma Relapsed non hodgkin lymphoma Neurotoxicity Neurotoxicity syndromes cytokine release syndrome	Drug: Anakinra Drug: Axicabtagene Ciloleucel	Massachusetts General Hospital Boston, Massachusetts, United States Dana Farber Cancer Institute Boston, Massachusetts, United States	Phase 2

In addition to the use of cytokine antagonists or neutralizing antibodies to treat CRS by ameliorating inflammation, some novel ideas and methods have recently been developed. In a study by Staedtke, a self-amplifying catecholamine loop in macrophages was revealed to have a critical role in CRS pathogenesis. In vivo analysis showed a noticeable reduction in tissue damage and inflammation in mice injected with bacteria that spontaneously secrete atrial natriuretic peptide (ANP). The same results were observed in the CD19 CAR T cell model, in which the release of catecholamines and cytokines resulting from the CAR T cell-tumor interaction can be inhibited by ANP administration. According to their report, the production of catecholamines in macrophages was TH-dependent, and MTR was confirmed to ameliorate inflammation-associated toxicities in CD19 CAR T cell-induced CRS, similar to that of ANP [23]. The innovative work by Staedtke has prompted us to identify other pathways that can regulate or activate immune cells or bystander cells and lead to cytokine release, which in turn provides a new means to block them and control CRS-associated toxicities.

Novel work from Karson S. Putt and Philip S. Low identified a new method for controlling CRS-associated toxicities. A low molecular weight adaptor, fluorescein-folate (FITC-folate) bridge, that specifically links CAR T cells and cancer cells was constructed. The FITC-folate bridge mediates the engagement of an anti-fluorescein CAR T cell with a folate receptor-expressing cancer. Due to the short circulation time, the number of adapter-mediated bridges between a CAR T cell and cancer cell can rapidly decline upon interruption of adapter administration, which enables reasonable control over the activation state and cytokine release activity of CAR T cells. It was also revealed that a temporary discontinuation of adapter administration could decrease cytokine-induced toxicities without compromising antitumor activity in NSG mice [50]. The use of this novel bispecific adapter provides a potential strategy for avoiding life-threatening CRS and a new vision for novel modifications of immunological synapses that form in the CAR T-tumor engagement system.

### Advantages or difficulties of current murine models that cannot meet the demand

When launching studies on systemic disorders such as CRS, suitable in vivo models are prerequisites. Since CRS occurs frequently in those with high tumor burdens, an in vivo model to first generate a basic leukemia tumor model and then infuse it with CAR T cells to initiate CRS is required. There are problems in developing models to mimic CRS in vivo. (i) Murine immunodeficiency is preferred when constructing a tumor in vivo model. On the other hand, CRS is an overreaction of the immune system, which means that an immunodeficient mouse might not be able to recapitulate the scenario occurring in patients with CRS after CAR T cell treatment. (ii) The species differences between humans and mice results in different immune system compositions, especially regarding some cytokines. (iii) To simulate CRS, we implanted tumor blasts and infused CAR T cells when the tumor model was established. Another challenge is whether we should apply human-derived leukemia cells or murine cell lines when establishing the tumor. Moreover, are human-derived CAR T cells or murine-derived CAR T cells preferable when generating the model?

Nevertheless, the current focus in CRS research is on the core cytokine-based mechanism of CRS pathogenesis, and the widely used human-derived CAR T cell and NSG mouse model is qualified for this work, to a certain extent. Since the different origins of CAR T cells and bystander cells can give us a huge advantage, we can have a way to determine the source of the cytokines, whether they are from CAR T cells or from bystander cells.

In addition, some researchers have noticed more severe CRS in SCID-beige mice than in NSG mice due to the different genetic backgrounds of SCID-beige and NSG mice [10]. NSG mice lack the common γ_c_ chain receptor that leads to impaired signaling of IL-2, IL-15 and other cytokines. Additionally, a deficient IL-1 response to IFN-γ or lipopolysaccharide (LPS) stimulation occurs in NSG mice [51]. It is beneficial that differences in the genetic backgrounds of mice can provide hints that might explain the involvement of different populations of immune cells in the CAR T cell-tumor interaction, such as the development and maturation defects of monocytes and macrophages in NOD mice, suggesting a reduction in macrophage reactivity and inhibited CRS in NSG mice [52].

In summary, the in vivo murine model cannot fully predict the clinical behavior of CAR T cells and CRS conditions, but it still has advantages in exploring the mechanism of CAR T cell-induced CRS.

## Conclusions

CRS has become one of the major barriers to applying CAR T cell therapy in tumors. Understanding the mechanisms of CRS pathogenesis is necessary for the development of novel CRS treatments. In the present review, we summarized novel progress in the field of CAR T cell therapy in the context of CRS and the involvement of macrophages in CRS pathogenesis. Several crucial CRS processes involve macrophages, including the initial activation of macrophages by the CD40L-CD40 interaction in the CAR T cell-tumor environment, the secretion of core cytokines (IL-6, IL-1 and IFN-γ) in CRS and a catecholamine self-amplification loop in macrophages. For the first time, we propose that CRS-associated toxicity might be a macrophage-centered pathophysiological process.

Presently, tocilizumab and corticosteroids are thought to be optional therapies to ameliorate CRS, according to NCCN guidelines and the clinical experiences of physicians. Since the critical role of macrophages in CRS has been reported in the latest research, we also summarized several potential macrophage-centered therapies that might be optimal treatments for CAR T cell-related CRS. Treatments blocking GM-CSF [22] and ANP/MTR [23] showed some efficacy in ameliorating CRS but did not affect CAR T cell functions. The innovative work of advanced engineering of FITC-folate bridges [50] in CARs that make CAR-tumor interactions controllable show huge potential. In general, the possible core role of macrophages in CRS provide us with a better understanding of CRS and might broaden the field of CRS treatment.

## Data Availability

Not applicable.

## Abbreviations

CAR: Chimeric antigen receptor
CRS: Cytokine release syndrome
IFN: Interferon
GM-CSF: Granulocyte-macrophage Colony Stimulating Factor
ANP: Atrial natriuretic peptide
TLR: Toll-like receptor
TNF: Tumor necrosis factor
SCID: Severe combined immunodeficiency
NOD: Non-obese diabetic
NSG: NOD/SCID/IL2rynull
iNOS: Inducible nitric-oxide synthase
